# Lessons on the Anatomy, Physiology and Hygiene of Infancy and Childhood

**Published:** 1901-05

**Authors:** 


					﻿Lessons on the Anatomy, Physiology and Hygiene of In-
fancy and Childhood.—For junior students, consisting of ex-
tracts from lectures given at Rush Medical College. By Alfred
C. Cotton, A. M., M. D. 174 pages. Price, $1.50. Chicago
Medical Book Co., 35 Randolph street, Chicago. 1900.
The first twenty pages of this book are devoted to the anatomy
of the new-born. The author draws attention to the fact that in-
fancy being the transitional stage, “the functions that minister to
growth are widely different from those similarly named in the
adult,” and that the disorders of infancy are oftenest due to a
want of symmetrical development, and to the neglect of the com-
monest principles of hygiene.
About twenty-five pages are given to the subject growth and
those important details so necessary to the proper appreciation of
the child are discussed in a clear and practical manner.
The physiology and hygiene of the new-born, including nour-
ishment up to one or two years, is perhaps the best part of the
book. About fifty pages are devoted to this part of the subject.
Then follow about sixty pages given to the discussion of milk,
weaning and substitute feeding, foods, the physiology and hygiene
of childhood, and the physiologic abnormalities of the new-born.
T. J. B.
				

